# Patient-reported outcomes in patients with hematological relapse or progressive disease: a longitudinal observational study

**DOI:** 10.1186/s12955-021-01887-6

**Published:** 2021-11-04

**Authors:** Mia Sommer, Lene Kongsgaard Nielsen, Lars Børty Nielsen, Rasmus Froberg Brøndum, Marlene Maria Nielsen, Anne Stoffersen Rytter, Charles Vesteghem, Marianne Tang Severinsen, Tarec Christoffer El-Galaly, Martin Bøgsted, Mette Grønkjær, Lone Jørgensen

**Affiliations:** 1 Department of Hematology, Aalborg University Hospital, Aalborg, Denmark; 2 Clinical Nursing Research Unit, Aalborg University Hospital, Aalborg, Denmark; 3 Department of Clinical Medicine, Aalborg University, Aalborg, Denmark; 4 Clinic for Surgery and Cancer Treatment, Aalborg University Hospital, Aalborg, Denmark; 5 Clinical Cancer Research Centre, Aalborg University Hospital, Aalborg, Denmark; 6 Quality of Life Research Centre, Department of Hematology, Odense University Hospital, Odense, Denmark; 7 Department of Internal Medicine and Cardiology, Regional Hospital Viborg, Viborg, Denmark; 8 University College Northern Denmark, Aalborg, Denmark

**Keywords:** Hematological cancer, Relapse, Patient-reported outcome, Precision medicine, Health-related quality of life

## Abstract

**Background:**

Patients with hematological cancer who experience relapse or progressive disease often face yet another line of treatment and continued mortality risk that could increase their physical and emotional trauma and worsen their health-related quality of life. Healthcare professionals who use patient-reported outcomes to identify who will have specific sensitivities in particular health-related quality of life domains may be able to individualize and target treatment and supportive care, both features of precision medicine. Here, in a cohort of patients with relapsed or progressive hematological cancer, we sought to identify health-related quality of life domains in which they experienced deterioration after relapse treatment and to investigate health-related quality of life patterns.

**Method:**

Patients were recruited in connection with a precision medicine study at the Department of Hematology, Aalborg University Hospital. They completed the European Organization for Research and Treatment of Cancer questionnaire and the Hospital Anxiety and Depression Scale at baseline and at 3, 6, 9, and 12 months after the relapse diagnosis or progressive cancer. Modes of completion were electronically or on paper. Clinically relevant changes from baseline to 12 months were interpreted according to Cocks’ guidelines. We quantified the number of patients with moderate or severe symptoms and functional problems and the number who experienced improvements or deterioration from baseline to 12 months.

**Results:**

A total of 104 patients were included, of whom 90 (87%) completed baseline questionnaires and 50 (56%) completed the 12-month assessments. The three symptoms that patients most often reported as deteriorating were fatigue (18%), insomnia (18%), and diarrhea (18%). The three functions that patients most often reported as deteriorating were role (16%) and emotional (16%) and cognitive (16%) functioning.

**Conclusion:**

In this study, patient-reported outcome data were useful for identifying negatively affected health-related quality of life domains in patients with relapsed or progressive hematological cancer. We identified patients experiencing deterioration in health-related quality of life during treatment and characterized a potential role for patient-reported outcomes in precision medicine to target treatment and supportive care in this patient group.

**Supplementary Information:**

The online version contains supplementary material available at 10.1186/s12955-021-01887-6.

## Background

Hematological cancers include non-Hodgkin lymphoma, Hodgkin lymphoma, chronic and acute leukemia, and multiple myeloma [1]. Globally, the incidence rate of hematological cancers has grown in the past decade [2], including a 39% rise in incident cases of non-Hodgkin lymphoma between 2007 and 2017 [2]. As a result of these increases, which mainly are attributable to population growth and aging [2–5], greater numbers of patients face the consequences of diagnosis, treatment, and subsequent relapses. Novel treatments have contributed to a more chronic nature of disease for some hematological cancers [2–5], and survival rates differ among them [6, 7]. For example, in diffuse large B-cell lymphoma, the most common subtype of non-Hodgkin lymphoma, the 5-year survival rate is 50%–60% during first-line treatment, and survival decreases with relapse [7].

Using HRQL as a clinical outcome has come into focus during the last decades, as an important parameter in understanding the impact of cancer treatment [8, 9].

HRQL can refer to different concepts and definitions, therefore, in the context of this study, the concept of HQOL is based on Wilson and Clearys conceptual model as an interrelation between biological, physical, social and psychological parameters linking clinical parameters, paraclinical data and health-related quality of life [10].

Studying the HRQL litterature in patients with hematological cancer, we found a systematic review from 2016 covering HRQL in this group of patients, only two included hematological patients with relapse. [11], Patients with relapse of acute leukemia and highly malignant lymphoma reported a worse HRQL compared to patients without relapse [12]. In addition, patients with relapse were significantly more fatigued and in pain [12]. The importance of HRQL studies in hematological patients with relapse is evident because these patients face yet another line of treatment and an even greater threat to life, which could potentially increase their physical and emotional trauma and worsen their HRQL.

The overall goal in precision cancer medicine is to match patients to treatments with higher efficacy based on individual genetic profiles [13]. At the Department of Hematology, Aalborg University Hospital, Denmark researchers have conducted a prospective and non-interventional population-based clinical study (ProGen/ProSeq) with the aim of describing genetic alterations in tumors from patients with hematologic relapse and to explore the potential of precision medicine [14].

In the context of precision medicine, the argument has been that healthcare professionals who can identify patients with specific sensitivities in particular HRQL domains will be better positioned to individualize and target treatments and supportive care. A way to assess HRQL domains is to elicit the information directly from the patient themselves using patient reported outcome (PRO) measures [15]. Early identification of patients with hematological cancer who experience deterioration in HRQL during relapse treatment could enable health professionals to consider alternative treatment options, adjust supportive care initiatives, or introduce psychosocial support to prevent further HRQL impairment. For these reasons, the aim of this study was to identify the HRQL domains most negatively affected after treatment and to investigate HRQL patterns in a cohort of patients with hematological cancer that had relapsed or progressed.

## Material and methods

### Study design

This population-based, longitudinal, observational HRQL survey study is a sub-study of the ProGen/ProSeq clinical study [14]. The aim of ProGen/ProSeq was to present the hematological precision medicine workflow developed at Aalborg University Hospital in a prospective, consecutively, and therefore unbiased population-based non-interventional study. We are reporting the results of this study based on the STROBE guidelines for observational studies [16].

### Setting and study population

Patients were recruited at the Department of Hematology, Aalborg University Hospital, in connection with scheduled diagnostic testing by study nurses affiliated with ProGen/ProSeq. Patient recruitment was conducted between March 2017 and November 2018, and data were collected between March 2017 and November 2019. Eligible patients were age ≥ 18 years, diagnosed with relapsed or progressing hematological cancer, and included in ProGen/ProSeq [14]. Exclusion criteria were non-verified relapse or progression of hematological malignant disease. The study was approved by the local ethics committee (N-20150042) as well as the data protection agency (2008-58-0028). The patients signed an informed consent before entering the study. The recruitment of patients and workflow for ProGen/ProSeq have been reported in detail elsewhere [14].

### Data collection: questionnaires

HRQL domains were assessed using the PRO instruments the European Organization for Research and Treatment of Cancer (EORTC QLQ-C30) and Hospital Anxiety and Depression Scale (HADS) questionnaires [17, 18]. EORTC QLQ-C30 is a validated instrument covering 15 multi-item and single-item domains of cancer [19]. It includes five functional domains (physical functioning, role functioning, emotional functioning, cognitive functioning, and social functioning), nine symptom scales (fatigue, nausea/vomiting, pain, dyspnea, insomnia, appetite loss, constipation, diarrhea, and financial difficulties), and one global health status (GHS) scale. Each scale is scored from 0 to 100, and a higher score indicates better GHS, better functioning, or more severe symptoms, respectively [20]. HADS is a symptom-specific questionnaire that measures anxiety and depression in patients with somatic disease. It consists of two subscales with 7 items each, one measuring anxiety and one measuring depression, which are scored separately [18].

The cut-off point for data collection was set to 12 months based on the study team’s judgement on when the effects of relapse would affect HRQL. Patients completed the questionnaires at baseline and at 3, 6, 9, and 12 months. Baseline was defined as the time point when patients received a verified test result of relapse or progressive disease. They could choose between completing the questionnaires electronically or on paper. Electronic questionnaires were distributed online via the clinical research database REDCap [21, 22]. Patients received an email containing a link to use to access the questionnaires, and the data were collected and stored in REDCap. A reminder was forwarded 1 and 2 weeks from the first invitation in case of non-response. Patients who chose to complete the questionnaires on paper received them by post, along with a prepaid return envelope. They did not receive reminders because we considered that the postal delivery time would affect real-time data collection. The responses to the returned questionnaires were entered into REDCap and double-checked by the first author. Non-response to baseline invitations was viewed as withdrawal of consent, and these patients were excluded from the study. Subsequent relapse during the study period was considered a clinical event that could influence HRQL and also led to exclusion from further PRO assessments.

### Data collection: demographic and clinical information

In addition to collection of PRO data, we extracted the following clinical data from electronic health records (EHRs): sex, age, diagnosis, Charlson’s comorbidity index score (CCI) [23], marital status and employment situation. The number of relapses were determined by review of EHR data and pathology reports. At baseline, the treating physicians were asked to report whether the initiated treatment was intended to be curative or non-curative and to estimate the patient’s survival prognosis as more than or less than 2 years. We also conducted an EHR review to collect date of diagnosis with advanced cancer and cancer type.

### Statistical analysis

The HRQL domain scores were calculated according to the questionnaire manuals [20, 24]. Missing items for EORTC-QLQ-C30 were handled according to the guidelines prescribed by the questionnaire developers [20]. Guidelines for missing items were not provided by the developers of HADS, so statistical handling of any missing items in HADS was according to guidelines by Bell et al. [25]. Patient characteristics, baseline scores, and response and dropout rates are presented using descriptive statistics.

### Patient-level analyses

We calculated the proportions of patients reporting moderate and severe symptoms or functional problems at baseline and at 12 months of follow-up. The thresholds for moderate and severe symptoms and functional problems are presented in Table 1. This grading of symptoms and functional problems has not been validated, however it has been applied in previous research in patients with hematological cancers [26–28]. For HADS, the thresholds were adapted from the developer’s scoring manual [24].

**Table 1:** Thresholds for moderate and severe symptoms and functional problems in EORTC-QLQ-C30 and HADS

	Moderate	Severe
*EORTC-QLQ-C30*		
Functional problems	>34 to < 67	≤ 34
Symptoms	>33 to < 66	≥ 66
*HADS*		
Anxiety and depression	> 10 to<15	≥﻿15

EORTC, European Organization for Research and Treatment of Cancer; HADS, Hospital Anxiety and Depression Scale

To identify the number of patients with an improvement or deterioration in HRQL, anxiety, or depression, we used responder analysis to evaluate each patient’s individual score change from baseline to 12 months of follow-up [29]. A responder in an EORTC-QLQ-C30 domain was defined as a patient reporting a reduction from severe to moderate or from moderate to mild in symptoms or functional problems, based on the defined thresholds. A responder in a HADS domain was a patient reporting a change in score that moved from one level to another, e.g., from moderate anxiety or depression to mild anxiety or depression [24]. Only patients completing the 12-month follow-up questionnaires were included in the responder analysis. We used Fisher’s exact tests to evaluate the hypothesis of no association between each HRQL domain and each of the following: age ≥ 70 years, CCI ≥ 5, curative-intent treatment, living alone, and estimated survival ≤ 2 years.

### Group-level analyses

The PRO data were modeled as outcomes in a linear mixed model to handle multiple responses from the same patient, with a random intercept per individual and age, sex, and survey time as fixed effects. Survey time was included as a categorical variable with the levels of baseline and 3, 6, 9, and 12 months. The mean change from baseline to the 12-month follow-up was tested for statistical significance and the clinical relevance was determined as minimally important difference (MID) and interpreted as trivial, small, medium, or large improvement or deterioration over time as described in Cocks’ guidelines for MID [30]. MID criteria for the HADS domains are not available for patients with cancer. For the anxiety and depression domains, we used distribution-based MIDs as thresholds for clinically meaningful change based on the standard error of measurement [31]. A *P* < 0.05 was considered statistically significant. The mean change in scores was considered evident if they were both clinically relevant and statistically significant. All statistical analyses were performed in R [32] using the implementation of Fisher’s exact test in the R-package *exact2* × *2* [33] and the R-package *lme4* [34] for linear mixed modeling, with *P* values for individual coefficients obtained with the R-package *lmerTest* [35]*.*

## Results

### Patient population and questionnaire completion

**Fig. 1:**
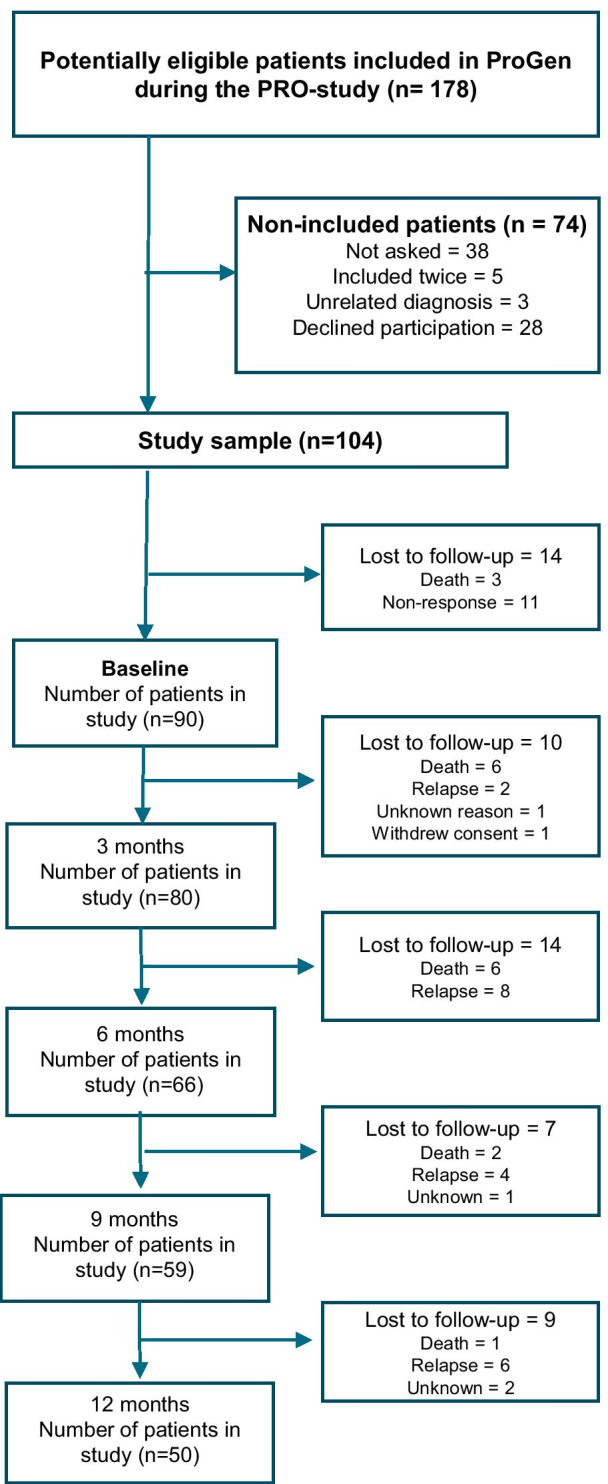
CONSORT flowchart of the inclusion process in a longitudinal observational study of health-related quality of life, anxiety, and depression

The inclusion process is illustrated in the CONSORT flow diagram in Fig. 1. In total, 178 patients were potentially eligible for the study, and 104 (58%) ultimately were included. Reasons for non-participation were ‘not asked’ (n = 38), double inclusion (n = 5), unrelated diagnoses (n = 3), and declined participation (n = 28). Overall, 14/104 (13%) patients did not complete the baseline questionnaires for unknown reasons (n = 11) or death (n = 3). Patients who did not complete baseline questionnaires for unknown reasons (n = 11) did not differ in age and diagnosis from those who did complete them; however, more men (8/11; 73%) than women did not complete the questionnaires at baseline.

The final group of participants (n = 90) included 60 (67%) men, and the median age was 69.5 (range 34–93) years. Additional clinical and sociodemographic characteristics are presented in Table 2. During the study period, 40 (44%) patients left the study. The most prevalent reason for dropout was subsequent relapse (n = 20; 50%), one patient (2%) withdrew consent, four (10%) dropped out for unknown reasons, and there were 15 deaths (38%). Thus, full data were missing for 40 out of 90 cases and intermittent data were missing for 8 of 342 questionnaires sent (2.3%).

**Table 2:** Clinical and sociodemographic characteristics of the included patients with hematological relapse

Characteristics	Total (%) N = 90 (100)
*Sex*	
Female	30 (33.3)
Male	60 (66.7)
*Median age, years*	69.5 (range, 34–93)
*Age*	
Age < 70	45 (50)
Age ≥ 70	45 (50)
*Diagnosis*	
Acute leukemia^¥^	9 (10)
Aggressive lymphoma^¶^	26 (28.9)
Chronic leukemia^**ǂ**^	18 (20)
Indolent lymphoma^§^	22 (24.4)
Multiple myeloma	15 (16.7)
*Treatment intent*	
Curative	19 (21.1)
Non-curative	71 (78.9)
*Expected survival*	
≤ 2 years	25 (27.8)
> 2 years	65 (72.2)
*Charlson’s Comorbidity Index*	
0–2	33 (36.7)
3–4	40 (44.4)
5+	17 (18.9)
Number of relapse/progressions*	
1	57 (63.3)
2	18 (20)
3+	15 (16.7)
*Marital status*	
Co-habiting	72 (80)
Single	18 (20)
*Employment status*	
Employed	19 (21.1)
Unemployed/retired	68 (75.6)
N/A	3 (3.3)
*Mode of completion*	
Electronic	70 (77.8)
Paper	20 (22.2)

^¶^Aggressive lymphomas included: diffuse large B-cell lymphoma, Hodgkin lymphoma, non-Hodgkin lymphoma, T-cell large granular lymphocytic leukemia, angioimmunoblastic T-cell lymphoma

^ǂ^Chronic leukemias included: chronic lymphocytic leukemia, small lymphocytic leukemia, chronic myeloid leukemia, hairy cell leukemia

^§^Indolent lymphomas included: follicular lymphoma, maltoma, mantle cell lymphoma, nodal marginal zone lymphoma, lymphoplasmacytic lymphoma, Waldenström’s macroglubulinemia, splenic marginal zone lymphoma, peripheral T-cell lymphoma

^¥^Acute leukemias included: Acute myeloid leukemia, acute lymphoid leukemia, myelodysplastic syndrome

^*^Including the current relapse/progression

We calculated the mean baseline scores and standard deviations for the total cohort, which are presented in Table 3.

**Table 3:** Mean baseline questionnaire scores for the total cohort

Health-related quality of life domains	Mean scores at baseline (± SD) (N = 90)
*EORTC-QLQ-C30*	
Global health status	59.3 (± 24.0)
Physical functioning	78.1 (± 21.0)
Role functioning	64.8 (± 30.7)
Emotional functioning	79.3 (± 18.2)
Cognitive functioning	83.5 (± 18.4)
Social functioning	80.1 (± 23.6)
Fatigue	40.6 (± 26.7)
Nausea and vomiting	8.7 (± 16.8)
Pain	23.9 (± 26.9)
Dyspnea	17.6 (± 23.1)
Insomnia	25.5 (± 29.7)
Appetite loss	20.0 (± 29.9)
Constipation	9.7 (± 20.2)
Diarrhea	16.3 (± 26.6)
Financial difficulties	4.5 (± 13.5)
*HADS*	
Anxiety	4.7 (± 3.2)
Depression	3.5 (± 3.0)

N, number of patients; SD, standard deviation; EORTC, European Organization for Research and Treatment of Cancer; HADS, Hospital Anxiety and Depression Scale. Each EORTC domain is scored from 0 to 100, and a higher score indicates better global health status, better functioning, or more severe symptoms, respectively. For HADS, the possible scores range from 0 to 21 for both scales, and a low score indicates a low level of anxiety or depression

### Changes at 12 months in moderate/severe symptoms and functional problems

In the total cohort, 41/90 (46%) reported moderate problems in GHS, 40/90 (44%) reported moderate fatigue, and 31/90 (34%) moderate problems in role functioning at baseline. Furthermore, 23/90 (26%) reported severe problems in role functioning, 20/90 (22%) reported severe fatigue, and 19/90 (21%) reported severe insomnia. Additional proportions of patients reporting moderate and severe symptoms or functional problems at baseline are presented in Table 4. Using the total sample of complete cases at the 12-month follow-up (n = 50), we found both deterioration and improvement from baseline in all HRQL domains. The three symptoms that most patients reported as deteriorating were fatigue (18%), insomnia (18%), and diarrhea (18%). The three functions that most patients reported as deteriorating were role (16%) , emotional (16%) and cognitive (16%) functioning. The proportions of patients with deterioration and improvement in functional and symptom domains are presented in Table 4. For a detailed plot of changes from baseline to 12 months please refer to Additional file 1.

**Table 4:** Baseline moderate-severe symptoms or functional problems and changes at 12 months for the full cohort at both time points

Health-related quality of life domains	Symptoms and functional problems at baseline, n (%) (N = 90)			Changes from baseline to 12 months, n (%) (N = 50)		
	Mild or no symptoms	Moderate	Severe	No change	Deterioration	Improvement
*EORTC-QLQ-C30*						
Global health status	30 (33)	**41 (46)**	19 (21)	30 (59)	7 (14)	**13 (27)**
Physical functioning	60 (70)	20 (22)	7 (8)	37 (74)	5 (10)	8 (16)
Role functioning	36 (40)	**31 (34)**	**23 (26)**	28 (56)	8 (16)	**14 (28)**
Emotional functioning	64 (71)	23 (26)	3 (3)	34 (68)	8 (16)	8 (16)
Cognitive functioning	66 (74)	20 (22)	4 (4)	36 (72)	8 (16)	6 (12)
Social functioning	57 (63)	25 (28)	8 (9)	36 (72)	3 (6)	11 (22)
Fatigue	30 (34)	**40 (44)**	**20 (22)**	28 (56)	**9 (18)**	13 (26)
Nausea and vomiting	79 (88)	8 (9)	3 (3)	44 (88)	2 (4)	4 (8)
Pain	50 (56)	28 (31)	12 (13)	35 (70)	4 (8)	11 (22)
Dyspnea	52 (57)	30 (34)	8 (9)	38 (76)	5 (10)	7 (14)
Insomnia	45 (50)	26 (29)	**19 (21)**	29 (58)	**9 (18)**	12 (24)
Appetite loss	55 (62)	22 (24)	13 (14)	27 (54)	8 (16)	**15 (30)**
Constipation	71 (79)	12 (13)	7 (8)	39 (78)	6 (12)	5 (10)
Diarrhea	60 (67)	19 (21)	11 (12)	33 (66)	**9 (18)**	8 (16)
Financial difficulties	80 (89)	8 (9)	2 (2)	43 (86)	3 (6)	4 (8)
*HADS*						
Anxiety	89 (99)	1 (1)	NA	44 (87)	5 (11)	1 (2)
Depression	89 (99)	1 (1)	NA	48 (96)	1 (2)	1 (2)

N, number of patients; NA, not applicable; SD, standard deviation; EORTC, European Organization for Research and Treatment of Cancer; HADS, Hospital Anxiety and Depression Scale

The three highest estimates for moderate and severe symptoms/functional problems and improvement/deterioration are marked in bold

To identify potential factors that could explain deterioration from baseline to 12 months, we tested selected baseline characteristics relative to HRQL domains. We found a possible statistically significant association between deterioration in RF and an expected survival ≤ 2 years at baseline, as estimated by the treating physician (odds ratio 0.14, 95% confidence interval 0.02–0.95, *P* = 0.04). Results of the regression analyses are presented as part of supplementary material (see Additional file 2).

### Changes in HRQL scores from baseline to 12 months

The clinically relevant changes in mean score and statistical significance after 12 months are shown in Table 5. In the total cohort, we found a small clinically relevant improvement at 3 months in GHS (*P* = 0.04), which was consistent at 6 months (*P* = 0.02), 9 months (*P* = 0.01), and 12 months (*P* = 0.04). We also found clinically relevant and statistically significant improvements in pain at 6 months (*P* = 0.01) and 9 months (*P* = 0.01), but the statistical significance faded at 12 months. Furthermore, at 9 months, we identified clinically relevant improvements in emotional functioning (*P* = 0.01) and fatigue (*P* = 0.03).

**Table 5:** Estimated mean changes (95% confidence intervals) in scores from baseline to 3, 6, 9, and 12 months

Health-related quality of life domains	3 months	*P*	Size, direction	6 months	*P*	Size, direction	9 months	*P*	Size, direction	12 months	*P*	Size, direction
*EORTC-QLQ-C30*												
Global health status	0.74 (− 4.48 to 5.96)	0.78	Trivial	6.61 (1.06–12.16)	**0.02**	**Small improvement**	7.66 (1.84–13.48)	**0.01**	**Small improvement**	6.39 (0.33–12.45)	**0.04**	**Small improvement**
Physical functioning	− 2.39 (− 6.21 to 1.44)	0.21	Trivial	0.22 (− 3.85 to 4.28)	0.92	Trivial	− 0.34 (− 4.63 to 3.95)	0.87	Trivial	1.57 (− 2.87 to 6.00)	0.48	Trivial
Role functioning	1.52 (− 4.68 to 7.72)	0.62	Trivial	2.94 (− 3.64 to 9.51)	0.37	Trivial	5.20 (− 1.74–12.14)	0.13	Trivial	4.40 (− 2.77–11.57)	0.22	Trivial
Emotional functioning	2.51 (− 1.53 to 6.55)	0.21	Trivial	3.76 (− 0.53 to 8.06)	0.08	Trivial	5.62 (1.11–10.12)	**0.01**	**Small improvement**	2.26 (− 2.43 to 6.95)	0.34	Trivial
Cognitive functioning	− 3.21 (− 7.26 to 0.84)	0.11	Small deterioration	0.04 (− 4.28 to 4.36)	0.99	Trivial	− 2.15 (− 6.68 to 2.39)	0.34	Trivial	− 3.54 (− 8.26 to 1.18)	0.14	Small deterioration
Social functioning	− 1.49 (− 6.63 to 3.64)	0.56	Trivial	3.37 (− 2.09 to 8.83)	0.22	Small improvement	2.01 (− 3.71 to 7.74)	0.48	Trivial	5.18 (− 0.78–11.14)	0.08	Small improvement
Fatigue	0.88 (− 3.91 to 5.67)	0.71	Trivial	− 4.13 (− 9.22 to 0.95)	0.11	Small improvement	− 5.87 (− 11.24 to − 0.51)	**0.03**	**Small improvement**	− 4.83 (− 10.37 to 0.72)	0.08	Small improvement
Nausea and vomiting	1.61 (− 2.15 to 5.36)	0.39	Trivial	− 3.52 (− 7.47 to 0.44)	0.08	Small improvement	− 3.87 (− 8.03 to 0.29)	0.06	Small improvement	− 4.23 (− 8.53 to 0.06)	0.05	Small improvement
Pain	− 2.06 (− 7.32 to 3.20)	0.43	Trivial	− 7.42 (− 13.00 to − 1.84)	**0.01**	**Small improvement**	− 8.10 (− 13.98 to − 2.22)	**0.01**	**Small improvement**	− 4.68 (− 10.76 to 1.40)	0.12	Trivial
Dyspnea	4.22 (− 0.93 to 9.38)	0.10	Trivial	− 0.41 (− 5.88 to 5.06)	0.88	Trivial	5.15 (− 0.62–10.91)	0.08	Small deterioration	2.61 (− 3.35 to 8.57)	0.38	Trivial
Insomnia	5.59 (− 1.02–12.21)	0.09	Small deterioration	0.35 (− 6.65 to 7.36)	0.92	Trivial	− 0.74 (− 8.12 to 6.64)	0.84	Trivial	− 3.77 (− 11.41 to 3.86)	0.32	Trivial
Appetite loss	− 2.41 (− 9.17 to 4.34)	0.48	Trivial	− 3.24 (− 10.41 to 3.94)	0.37	Trivial	− 6.16 (− 13.68 to 1.36)	0.10	Trivial	− 5.56 (− 13.34 to 2.22)	0.15	Trivial
Constipation	2.59 (− 2.25 to 7.43)	0.29	Trivial	− 2.00 (− 7.13 to 3.12)	0.44	Trivial	− 0.70 (− 6.13 to 4.74)	0.80	Trivial	0.24 (− 5.34 to 5.82)	0.93	Trivial
Diarrhea	4.00 (− 1.86 to 9.86)	0.17	Trivial	− 4.33 (− 10.57 to 1.91)	0.17	Trivial	− 5.01 (− 11.55 to 1.54)	0.13	Small deterioration	− 2.62 (− 9.43 to 4.20)	0.44	Trivial
Financial difficulties	2.80 (− 0.97 to 6.58)	0.14	Trivial	0.27 (− 3.74 to 4.29)	0.89	Trivial	0.82 (− 3.38 to 5.03)	0.70	Trivial	0.92 (− 3.46 to 5.30)	0.67	Trivial
*HADS*												
Anxiety	0.02 (− 0.68 to 0.73)	0.95	No clinical change	− 0.08 (− 0.82 to 0.66)	0.83	No clinical change	− 0.76 (− 1.54 to 0.02)	0.05	No clinical change	0.15 (− 0.66 to 0.96)	0.72	No clinical change
Depression	− 0.27 (− 0.93 to 0.39)	0.41	No clinical change	− 0.55 (− 1.25 to 0.15)	0.12	No clinical change	− 0.65 (− 1.38 to 0.09)	0.08	No clinical change	− 0.56 (− 1.32 to 0.21)	0.15	No clinical change

N, number of patients; EORTC, European Organization for Research and Treatment of Cancer; HADS, Hospital Anxiety and Depression Scale; CI, confidence interval

Health-related quality of life domains with both a statistically significant and a clinically relevant change from baseline are marked in bold. Estimates are adjusted for effects of age and sex and with a random intercept per individual

## Discussion

In this study, we investigated HRQL patterns in a cohort of patients with hematological cancer during treatment for relapse or progressive cancer to 12 months later in the context of precision medicine. Our results demonstrate that the patients in general reported stable HRQL during relapse treatment. Individually, several patients reported moderate and severe symptoms and functional problems at the time of relapse. In addition, we found that between 16% and 18% of the patients experienced deterioration in insomnia, diarrhea, and fatigue, and that 16% experienced impaired role and impaired emotional and/or cognitive functioning at 12 months of follow-up. We identified patient and clinical characteristics associated with deterioration in HRQL during relapse or progressive cancer treatments and found that deterioration in role functioning was possibly associated with an estimated survival of less than 2 years.

### Results in a clinical context

It has been reported that patients with hematological cancers experience anxiety and depression [36, 37]. Studies also suggest the same effects on patients with hematological relapse [38, 39], which is not surprising given that they face yet another life-threatening situation. Fear of recurrence has been reported as a major concern for this patient population after treatment completion [36, 40, 41] and would be expected to lead to anxiety, as would relapse when it is diagnosed. However, we found low levels of both anxiety and depression at baseline as well as at the 12-month follow-up in our patients experiencing relapse. The cut-off for mild symptoms of either condition in HADS is  ﻿≤10, and the respondents in this study had mean baseline scores of 4.7 for anxiety and 3.5 for depression.

The analysis also illustrated a constant level of severe problems in role functioning from baseline to the 12-month follow-up, as well as a possible association between deterioration in role functioning at 12 months and an estimated survival ≤ 2 years. These results may point to a lack of support in this area, suggesting a gap for those in palliative care who are coping with carrying out daily activities. This argument is supported by the results of Ramsenthaler et al., who found that one of the most burdensome concerns in patients with multiple myeloma in palliative care was not being able to carry out daily activities [42].

For the total cohort, GHS, fatigue, and insomnia were the domains with proportionally high levels of moderate and severe symptoms and functional problems at baseline. The cohort on average experienced an improvement in the three domains from baseline to 12 months of follow-up, although a large proportion still reported moderate or severe symptoms and functional problems at 12 months. These results establish that this group of patients experiences a large symptom burden after the first year of relapse treatment. Our results are consistent with those of Johnsen et al., who found that among patients with mixed hematological cancer types, at baseline, 55% reported moderate fatigue symptoms and 46% reported moderate insomnia symptoms [27]. Furthermore, those authors found severe fatigue in 20% of the patients and severe symptoms of insomnia in 15% [27]. Taking together the current findings and previously published results, the implication is that some patients with hematological cancer experience persistent disease symptoms or symptomatic side effects from relapse treatment, in particular fatigue, insomnia, and reduced GHS. These HRQL domains should be especially monitored in clinical practice and addressed in a timely way to improve outcomes for these patients.

This study adds to the limited HRQL literature in patients with hematological relapse or progressive disease. To date, most such studies are conducted as part of clinical trials and thus based on highly selected populations. The present study was conducted in a population-based cohort and offers insight into the course of quality of life as seen in clinical practice.

### Potential role of PROs in precision medicine

Research is increasingly focused on the value of routine symptom monitoring and the role of PRO data in cancer care as a strategy for individualized medicine [43, 44]. Basch et al. reported that routine PRO monitoring promotes effective and individually tailored care as well as identifying both unnecessary visits and immediate care needs [43, 44].

In this study, we aimed to investigate HRQL patterns in a cohort of patients with hematological cancer that had relapsed or progressed. The reason for this exploration was that early identification of patients with hematological cancer who experience deterioration in HRQL during relapse or progressive cancer treatment may support clinical decision making regarding alternative treatment options, supportive care initiatives, or introduce psychosocial support to prevent further HRQL impairment [45]. Based on the group-level analyses, the patients in general reported stable HRQL during relapse treatments, but the patient-level analyses showed that some of them experienced HRQL deterioration in the 12 months after a diagnosis of relapse or progressive disease. Hence, some patients may not receive adequate support during treatment for relapse or progressive disease. Relapse treatment poses a risk of overtreatment in terms of yielding limited effects because of increased risk of therapy resistance [14]. Furthermore, clinicians are not always able to ensure improvements in or stabilization of HRQL. This dilemma poses a challenge in clinical decision-making for identifying which patients will not experience HRQL improvement or stabilization during treatment. Hence, in this study, we demonstrate that symptom management for some patients is not effective and that by introducing PRO data in clinical practice during relapse treatment may support clinical decision-making providing patients with targeted and individualized symptom management.

This study is limited by the fact that baseline was defined as the time point when patients received a relapse diagnosis, which is when the baseline questionnaires were forwarded. In case of non-response to the baseline questionnaires, the patients received reminders up to 2 weeks after the diagnosis. This delay could potentially mean that they started treatment before completing the first questionnaires, possibly resulting in biased baseline scores in either direction because of treatment causing or alleviating symptoms. Moreover, during the study period, only 50 patients completed the 12-month follow-up, representing a dropout rate of 56%. The dropout rate has undoubtedly limited the statistical power, which should be considered when interpreting the results. One of the main reasons for dropout was death, which may be expected given that these are patients with advanced disease. Finally, the primary endpoint for PRO data collection was set to 12 months and may have contributed to the amount of missing data and/or large drop-out and, hence, had the data collection cut-off point been 6 or 9 months the results may have been different.

Potentially informative but missing PRO data are a much-debated topic within longitudinal quality of life research. As recommended, we report the compliance rate and reasons for dropout [46], but no published valid statistical analysis strategy is available for managing informative missing PRO data to enhance the robustness of the findings [47]. In addition, 19 patients diagnosed with subsequent relapse during the study period were excluded. In hindsight, these patients should have continued, and a subsequent relapse could have been adjusted for in the statistical analysis [48]. Inclusion of these data may have improved the statistical power.

Study participants tend to be healthier than non-responders and dropouts [49], and patients whose treatment yields a favorable outcome may report better HRQL than those who experience a less favorable outcome. A subgrouping of the cohort into curative and non-curative treatment strategies would have been helpful for further exploration of this angle. With these assumptions, the results may be overestimated, which should be considered in interpreting them. However, this population may also suffer from even more severe symptoms and functional problems during relapse treatment than reported in this study. Overall, because of the small sample size and high dropout rate, the results of this study should be interpreted cautiously, but they may be considered preliminary findings that are useful for generating hypotheses for larger studies.

## Conclusion

This study adds to a limited evidence base on HRQL in patients with hematological cancer during their first year of treatment for relapse or progressive disease. At baseline, these patients seem to report moderate and severe symptoms and functional problems in most HRQL domains, and from the patient-level analysis, we identified domains in which they reported deterioration at 12 months. Although this study is limited because of the small sample size and high dropout rate, the analyses represent a model of how PRO data could be integrated into precision medicine for  clinical decision-making and supportive care management to improve outcomes for patients with relapse or progressive hematological cancers.

### Perspectives and future research

This study demonstrates that PRO data may be valuable in patients with relapse or progression of hematological cancer based on a longitudinal PRO data collection. However, we still do not know if and how PRO data can be effectively integrated and actively used to support real-time clinical decision-making in this patient group. Therefore, future studies on this topic are needed to further investigate PRO data’s supportive role in clinical decision-making and supportive care management in order to benefit patients and facilitate quality in care and treatment.

## Supplementary Information

**Additional file 1**. Changes from baseline to 12 months follow-up in EORCT-QLQ-C30 and HADS domains

**Additional file 2**. Association of baseline characteristics with deterioration risk by EORTC-QLQ-C30 and HADS domains

## Data Availability

The datasets used and/or analyzed during the current study are available from the corresponding author on reasonable request.

## Abbreviations

CCI: Charlson Comorbidity Index
CI: Confidence interval
EORTC: European Organization for Research and Treatment of Cancer
GHS: Global health status
HADS: Hospital Anxiety and Depression Scale
HRQL: Health-related quality of life
N: Number of patients
OR: Odds ratio
PRO: Patient-reported outcome
SD: Standard deviation
