# Supplementary figures and images for: Patient-reported outcomes in patients with hematological relapse or progressive disease: a longitudinal observational study
Source: Health Qual Life Outcomes. 2021 Nov 4;19:251. doi: 10.1186/s12955-021-01887-6 (PMC8567661; doi:10.1186/s12955-021-01887-6)

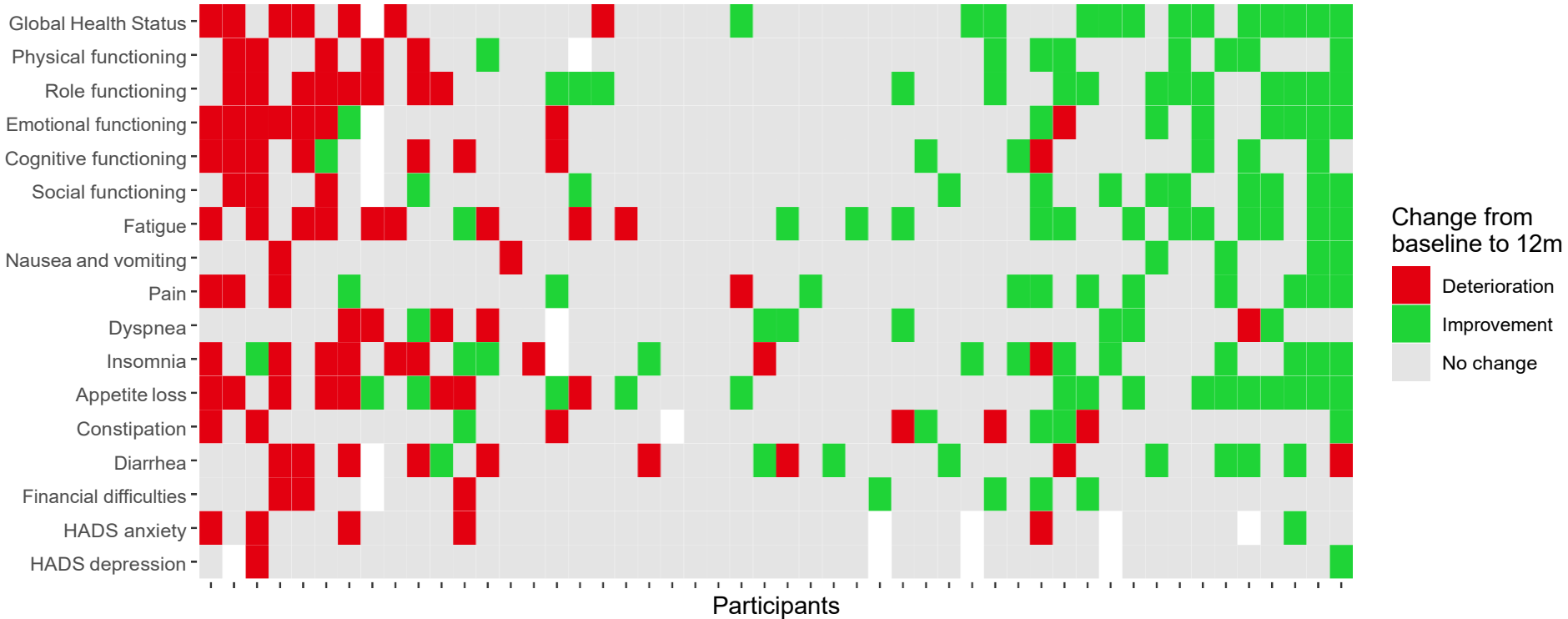

Supplement: Supplementary file 1 — Additional file 1. Changes from baseline to 12 months follow-up in EORCT-QLQ-C30 and HADS domains [file 12955_2021_1887_MOESM1_ESM.pdf]
