# Supplementary material for: Patient-reported outcomes in patients with hematological relapse or progressive disease: a longitudinal observational study
Source: Health Qual Life Outcomes. 2021 Nov 4;19:251. doi: 10.1186/s12955-021-01887-6 (PMC8567661; doi:10.1186/s12955-021-01887-6)
Supplement: Supplementary file 2 — Additional file 2. Association of baseline characteristics with deterioration risk by EORTC-QLQ-C30 and HADS domains [file 12955_2021_1887_MOESM2_ESM.docx]

| **Additional file 2: Association of baseline characteristics with deterioration risk by EORTC-QLQ-C30 and HADS domains.** | | | | | |
| --- | --- | --- | --- | --- | --- |
| **Health-related quality of life domains** | Age ≥70 years | CCI ≥5 | Curative-intent treatment | Living alone | Estimated survival ≤2 years |
|  | **OR (95% CI)** | **OR (95% CI)** | **OR (95% CI)** | **OR (95% CI)** | **OR (95% CI)** |
| **EORTC-QLQ-C30** | | | | | |
| GHS | 1.60 (0.31–9.06) | 0.63 (0.11–3.22) | 0.84 (0.03–7.93) | 0.89 (0.03–9.38) | 0.27 (0.04–2.5) |
| Physical functioning | 0.80 (0.09–5.66) | 1.36 (0.19–11.92) | 0 (0–4.72) | 1.40 (0.05–13.32) | 0.52 (0.05–14.86) |
| Role functioning | 0.67 (0.12–3.11) | 0.83 (0.18–3.92) | 0.72 (0.03–6.17) | 0.64 (0.02–5.52) | **0.14 (0.02–0.95)** |
| Emotional functioning | 0.64 (0.12–2.99) | 0.24 (0.03–1.34) | 0.70 (0.03–6.01) | 0.74 (0.03–7.09) | 0.33 (0.05–2.98) |
| Cognitive functioning | 0.13 (0.01–1.05) | 0.48 (0.09–2.24) | 1.91 (0.23–13.09) | 0 (0–3.08) | 0.33 (0.05–2.98) |
| Social functioning | 2.34 (0.17–71.7) | 0.43 (0.01–5.8) | 0 (0–9.23) | 0 (0–9.88) | 0.25 (0.02–8.46) |
| Fatigue | 0.93 (0.21–3.96) | 0.36 (0.07–1.78) | 1.65 (0.2–10.19) | 3.10 (0.52–20.98) | 0.39 (0.06–3.39) |
| Nausea and vomiting | 1.18 (0.03–47.19) | 0.85 (0.02–34.02) | 0 (0–18.9) | 0 (0–17.08) | N/A |
| Pain | 0.37 (0.01–3.62) | 0.26 (0.01–2.57) | 0 (0–6.32) | 0 (0–5.7) | N/A |
| Dyspnea | 0.73 (0.08–5.17) | 1.24 (0.18–10.91) | 4.05 (0.43–31.81) | 1.17 (0.04–10.84) | 0.16 (0.02–1.62) |
| Insomnia | 1.51 (0.35–6.83) | 0.6 (0.13–2.56) | 0 (0–2.28) | 0 (0–2.02) | 1.14 (0.11–29.86) |
| Appetite loss | 1.21 (0.25–5.71) | 0.83 (0.18–3.92) | 0.72 (0.03–6.17) | 0.64 (0.02–5.52) | 0.95 (0.09–25.1) |
| Constipation | 0.22 (0.01–1.89) | 0.15 (0.01–1.31) | 0 (0–4.55) | 2.75 (0.31–18.72) | 0.66 (0.07–18.32) |
| Diarrhea | 0.51 (0.1–2.48) | 0.66 (0.15–2.83) | 3.39 (0.57–22.79) | 0.63 (0.02–5.66) | 0.17 (0.02–1.15) |
| Financial difficulties | 0 (0–1.89) | 0.43 (0.01–5.8) | 12.18 (0.84–395.16) | 0 (0–9.88) | 0.25 (0.02–8.46) |
| **HADS** | | | | | |
| Anxiety | 0.24 (0.01–2.07) | 0.53 (0.06–3.75) | 1.77 (0.06–17.49) | 0 (0–3.83) | 0.13 (0.01–1.39) |
| Depression | 0 (0–20.73) | 0 (0–15.96) | 0 (0–105.86) | 0 (0–80.75) | 0 (0–2.85) |

N, number of patients; EORTC, European Organization for Research and Treatment of Cancer; HADS, Hospital Anxiety and Depression Scale; CCI, Charlson Comorbidity Index; OR, odds ratio; CI, confidence interval.

Statistically significant results, based on results of Fisher’s exact tests, are marked in **bold**.
